# Effects of Atmospheric Pressure Plasma Jet on 3D-Printed Acrylonitrile Butadiene Styrene (ABS)

**DOI:** 10.3390/ma17081848

**Published:** 2024-04-17

**Authors:** Andrei Vasile Nastuta, Mihai Asandulesa, Iuliana Spiridon, Cristian-Dragos Varganici, Ramona Huzum, Ilarion Mihaila

**Affiliations:** 1 Physics and Biophysics Education Research Laboratory (P&B-EduResLab), Biomedical Science Department, Faculty of Medical Bioengineering, “Grigore T. Popa” University of Medicine and Pharmacy Iasi, M. Kogalniceanu Str., No. 9–13, 700454 Iasi, Romania; 2 “Petru Poni” Institute of Macromolecular Chemistry, 41A Gr. Ghica Voda Alley, 700487 Iasi, Romania; 3 Integrated Center of Environmental Science Studies in the North-Eastern Development Region (CERNESIM), Department of Exact and Natural Sciences, Institute of Interdisciplinary Research, “Alexandru Ioan Cuza” University of Iasi, Blvd. Carol I No. 11, 700506 Iasi, Romania

**Keywords:** plasma jet, work of adhesion, plasma-treated ABS surface, surface characterization, AFM, mechanical properties

## Abstract

Polymers are essential in several sectors, yet some applications necessitate surface modification. One practical and eco-friendly option is non-thermal plasma exposure. The present research endeavors to examine the impacts of dielectric barrier discharge atmospheric pressure plasma on the chemical composition and wettability properties of acrylonitrile butadiene styrene surfaces subject to the action of additive manufacturing. The plasma source was produced by igniting either helium or argon and then adjusted to maximize the operational conditions for exposing polymers. The drop in contact angle and the improvement in wettability after plasma exposure can be due to the increased oxygen-containing groups onto the surface, together with a reduction in carbon content. The research findings indicated that plasma treatment significantly improved the wettability of the polymer surface, with an increase of up to 60% for both working gases, while the polar index increased from 0.01 up to 0.99 after plasma treatment. XPS measurements showed an increase of up to 10% in oxygen groups at the surface of He–plasma-treated samples and up to 13% after Ar–plasma treatment. Significant modifications were observed in the structure that led to a reduction of its roughness by 50% and also caused a leveling effect after plasma treatment. A slight decrease in the glass and melting temperature after plasma treatment was pointed out by differential scanning calorimetry and broadband dielectric spectroscopy. Up to a 15% crystallinity index was determined after plasma treatment, and the 3D printing process was measured through X-ray diffraction. The empirical findings encourage the implementation of atmospheric pressure plasma-based techniques for the environmentally sustainable manipulation of polymers for applications necessitating higher levels of adhesion and specific prerequisites.

## 1. Introduction

Polymers have a vital role in several sectors like biology, medicine, building, textiles, machinery, and the food industry. Nevertheless, there are instances where altering polymer properties (especially the surface properties) is essential before further material processing. This is due to the fact that polymers usually have certain surface properties that are considered insufficient for various specific applications. These materials are primarily utilized as crucial elements in a wide variety of mechanical, electrical, or biological components due to their major bulk physicochemical features. Nevertheless, the surface properties of the polymers are frequently critical, necessitating the meticulous identification and acquisition of these characteristics. Non-thermal plasma exposure is commonly used to alter the surface characteristics of polymers [1,2,3,4,5,6,7,8,9], even for biofunctionalizing 3D-printed structures [10].

Standard physical techniques used in technology to study how materials interact are not as helpful or good for the environment as plasmas at room temperature and pressure. Many reactive nitrogen and oxygen species (RNS/ROS) are present in the plasma–matter interaction. There are many areas where they are very important, such as food, medicine, the auto industry, the environment, and farming [11,12,13,14,15,16,17,18,19]. Another application in which plasma acts on polymers is its possible use in the ’repair of damaged polymer composites surfaces’, as reported by [20].

Many rigorous multidisciplinary research activities are being driven by new plasma–surface, plasma–liquid, and plasma–gas applications. Plasma has many potential uses in biomedicine, pharmacology, food science, bioengineering, agriculture (e.g., as an antibacterial, disinfectant, antiseptic, and wound healer), transportation, and seed germination. That said, plasma-based technologies are the ones that are covered in the aforementioned discussion of plasma research directions [21,22,23].

Polymers must be understood by gathering as much information as possible. Despite multiple published experiments, plasma–polymer surface interactions are still poorly understood, requiring continued investigation. Polymers’ surface morphology (through shape) and energy, as well as mechanical properties (like flexibility and coefficient of friction) and even interfacial tension coefficient, can be changed. Thus, understanding these characteristics helps in the development and improvement of new physicochemical methods to change them. Polymers can be functionalized, cleaned, etched, and crosslinked using atmospheric pressure non-thermal plasma [7,24,25,26].

Plasma–polymer interface processes are affected by electrical parameters, like voltage, current density, electrical power, or total radiation power, and by excited/reactive species. Thus, adjusting these parameters can alter plasma-induced polymer surface changes. Due to atmospheric pressure, the surface has many oxygen-containing chemical groups, such as hydroxyl, carbonyl, or carboxyl/ester. This is the case no matter what plasma working gas is used—oxygen, air, carbon dioxide, helium, or argon. Functional groups that contain oxygen are often found on the surface of the plasma-treated polymer because oxygen sticks very well to this surfaces. It is worth mentioning that the type of gas used to generate the plasma has a large effect, as was shown in experiments and stressed by Kumar et al. [27].

To fully grasp how polymers behave, one must comprehend the interactions that take place between the surfaces of these materials and plasma. Material engineers use additive manufacturing (AM) to generate three-dimensional scaffolds. Among the methods that fall under this category are stereolithography, fused deposition molding (FDM), and selective laser sintering (SLS) [28]. The most used additive manufacturing approach in electronics, architecture, and tissue engineering is extrusion-based printing (EBP).

Fluidic structures for biological applications and nanomaterials are among the many applications of additive manufacturing [29,30,31]. Multiple studies in the literature analyze how FDM process parameters affect the mechanical properties of printed polymers. These studies emphasize the importance of researching the impact of operational parameters of additive manufacturing on the final result [32,33,34,35].

Acrylonitrile butadiene styrene (known as ABS), is a thermoplastic that is amorphous and resistant to impact. Styrene, butadiene, acrylonitrile, and butadiene are its constituent monomers. However, it is extruded at high temperatures despite the fact that it is not biodegradable. Due to its outstanding physical qualities, including impact resistance and hardness, it is commonly selected for structural applications and frequently employed in FDM procedures. Moreover, as Zaldivar et al. have documented, one of the potential impacts of plasma on ABS is that it can potentially reinforce 3D-printed ABS that has been treated with plasma [36]. Devices such as computers, pipes, boats, and LEGO toys all make use of it [37].

The manufacturing industry has been completely transformed due to the development of additive manufacturing technologies, which enables the fabrication of intricate structures with an accuracy that is truly unparalleled. However, before designing 3D printing materials, wettability must be considered. Surface tension and moisture affect printing material adhesion and stacking, which affects final product quality and integrity.

This study examines the impact of a plasma source at atmospheric pressure, created by a dielectric barrier discharge (DBD) system with a cylindrical geometry using helium (He) and argon (Ar), on the surface properties, hydrophilicity, and chemical composition of acrylonitrile butadiene styrene (ABS) that needs to be 3D printed, as an alternative to other conventional polymer modification methods (e.g., chemical grafting).

## 2. Materials and Methods

### 2.1. Plasma Source and Material Preparation

#### 2.1.1. Plasma Source Set-up and Diagnosis Methods

**Figure 1:**
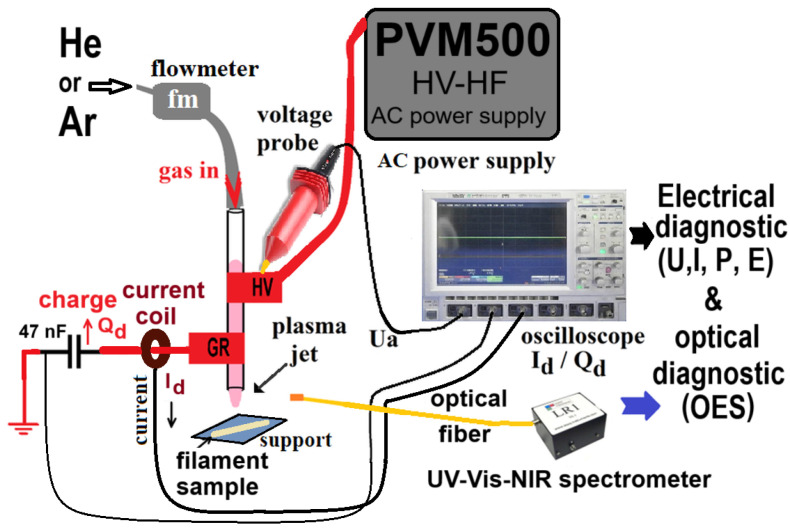
This experiment makes use of a plasma source with electrical and optical diagnostic instruments.

Figure 1 shows how a commercial ABS polymer fiber with a 1.75 mm diameter that is meant for 3D printing is treated using a plasma source. In the trials, feed gases He and Ar were used. The gases passed through a quartz discharge tube that was 100 mm long and had inner and outer diameters of 4 mm and 6.1 mm. The flow rate of the tube was 2.0 standard liters per minute. The 10 mm wide commercial adhesive copper tape, which was 0.5 mm in thickness, was used to make the electrodes with high voltage (HV) power and ground (GR) surrounding the exterior of the quartz tube. They were positioned outside the quartz tube, with a gap of 10 mm between them. Ten millimeters separated the ground electrode from the tube’s nozzle. In this experiment, the HV electrode was linked to the power source via a power connection, while the GR one was connected to the ground. This procedure used a technique similar to those described in references [38,39,40,41].

Plasma was ignited by a PVM500 power source (Information Unlimited, Amherst, MH, USA). This supply has the capability to produce voltages of up to 40 kV, with frequencies spanning from 20 to 70 kHz and an adjustable power output of 1 to 300 W. The sinusoidal voltage U${\;\;}_{a}$ was measured using a CT4028 high-voltage probe from Cal Test Electronics Inc. It reached a peak-to-peak value of 14 kV and had a frequency of 48 kHz, corresponding to an average power output of around 10 watts. The total discharge current I${\;\;}_{d}$ was monitored using a PP006A voltage probe from Lecroy, a high-precision high-frequency current transformer CT-D1.0-B from Magnelab, and a 47 nF charge capacitor, all of which were linked to a 1 GHz digital oscilloscope (Lecroy WaveSurfer 104Xs, Chestnut Ridge, NY, USA) with four channels and a sampling rate of 2.5 GS/s. The discharge power was determined by analyzing Lissajous figures created by the charges on the capacitor and the applied voltage during the discharge. The experiment utilized pure helium and pure argon (He 5.0 and Ar 5.0, Siad Romania, Bucharest, Romania), which were introduced into the discharge quartz tube at 2.0 standard liters per minute. A wide-range spectrometer (LR1 ASEQ, Vancouver, CA, is used to study the electromagnetic spectrum of the plasma in the UV-Vis-NIR (ultraviolet-visible near-infrared) region. The optical fiber, which was 1 m long and 400 μm in diameter, was placed side-on 5 mm away from the plasma effluent and 5 mm away from the discharge tube (nozzle) in order to record the 200 to 900 nm plasma emission spectra. The experimental setup depicted in Figure 1 has been thoroughly documented in previous literature [19,41,42,43]. Moreover, Huzum et al. [41], Burducea et al. [43], and Nastuta et al. [19,42] utilized a comparable setup for various purposes such as grape must treatment, wheat seed treatment, and plasma parameter optimization to establish proper plasma settings for the exposure of various surfaces.

#### 2.1.2. Material Preparation

For this study, there were two sets of samples, namely one consisting of untreated and plasma-treated ABS filaments and a second set consisting of 3D-printed ABS samples in the shapes required for certain analysis methods. The exposure time in both He and Ar–plasma was 5 min, with the filament being moved through the plasma effluent constantly to ensure a proper isotropic treatment. The plasma treatment was carried out only on the ABS filament samples, with the analysis being performed on both the filament (untreated and plasma-treated He/Ar) and on the 3D-printed objects (different geometries depending on the analysis method) by comparing objects made out of filament that were untreated and plasma-treated (in He/Ar). Before and after the plasma treatment, the set of samples of ABS filaments (1 cm long) were characterized by means of atomic force microscopy, contact angle (4 cm long), X-ray photo-electron spectroscopy, Fourier Transformed infrared spectroscopy, and X-ray diffraction. This study used commercially available white acrylonitrile butadiene styrene filament that was 1.75 mm in diameter (Devil Design Sp. J. in Milkolov, Poland). The second set consisted of several different 3D-printed ABS samples that were used for the tests, with the needed filament for these objects being untreated or plasma-treated, accordingly. More precisely, for surface and volume analysis, rectangular objects (10 × 10 mm${\;\;}^{2}$, 0.5 mm thick) were 3D printed using ABS filaments. For the dielectric tests, cylinders with a diameter of 10 mm and a height of 0.5 mm were printed. For the mechanical tests, dog-bone-shaped specimens, based on the standard ASTM D638 IV [44,45], were used for tensile tests. For impact tests, specimens were prepared and used according to EN ISO 179-1:2023 [46]. For these tests, a fused deposition modeling (FDM)-based MK3S+ 3D printer (Prusa Research a.s., Prague, Czech Republic) was used. The printer’s settings for printing ABS filament were as follows: a 0.4 mm nozzle heated to 255 °C, a bed heated to 100 °C, a layer height of 0.15 mm (PrusaSlicer software, v2.7.1), and an infill that was 100% rectilinear.

### 2.2. Surface and Volume Characterization Methods of Polymeric Sample

While many of the mechanical, electrical, and thermal properties of ABS have been extensively studied, there are a lack of data on this material’s surface characteristics in the literature. This is the case even though some of these attributes have been studied. Innovative surface characteristics, such as the biocompatibility of the material, are imperative for novel applications, including the utilization of additive manufacturing techniques for a variety of objectives. This is because new applications require the development of innovative surface qualities. Given that environmental preservation is one of society’s most pressing challenges, the plasma surface modification of these materials might prove effective. To investigate plasma exposure changes, polymeric samples were quantified by volume and surface properties using multiple methods.

#### 2.2.1. Surface Morphology: Atomic Force Microscopy

Atomic force microscopy, or AFM, is a powerful technique used to study how processing conditions, including plasma treatment, affect the surface morphology of polymers [40,47,48,49]. The polymeric samples were observed while in tapping mode and in air using a Solver Pro-M microscope (AFM, NT-MDT, Zelenograd, Russia) with an NSC21 cantilever (with a tip radius less than 10 nm, a resonance frequency centered at 201 kHz, and a force constant of 17.5 N/m, Mikromasch, Sofia, Bulgaria).

Besides three-dimensional topographical images and roughness parameters, expressed through root mean square roughness (S${\;\;}_{q}$), other statistically meaningful surface quantities, like the mean roughness value (S${\;\;}_{a}$), skew value (S${\;\;}_{sk}$), and excess kurtosis (ek), as well as the values of maximum peak height (S${\;\;}_{p}$), maximum pit depth (S${\;\;}_{v}$), and maximum height (S${\;\;}_{z}$), were also calculated using a dedicated image processing software (Gwyddion, v 2.64) [50].

#### 2.2.2. Static Contact Angle and Surface Energy

In order to determine the wettability of a material, two parameters must be evaluated: contact angle (CA) and its surface energy, as well as how surface changes (e.g., via plasma exposure) affect the wettability of polymers [14,51,52,53].

The value of the surface energy of ABS specimens has been quantified via the sessile drop method. A self-made apparatus, as referenced in [19,40], is utilized to measure and store images of liquid droplets. The CA system comprises a 2X lens goniometer with a 2 Mp digital camera and an LED as the light source. Droplets with a 1 μL volume of distilled water and glycerol were placed on the sample surfaces at room temperature, and photos of the droplets on the material were captured. Contact angle values were measured using ImageJ software v1.54d through the Drop Analysis plug-in, as reported by Schneider et al. [54] and Stalder et al. [55]. In the current experiment, the surface energy value (γ) was ascertained by examining the polar and dispersive components of glycerol and distilled water.

#### 2.2.3. X-ray Photoelectron Spectroscopy (XPS) of Polymers

The effects of discharge exposure on ABS samples (filament and 3D prints) were investigated using X-ray photoelectron spectroscopy (XPS). In order to maintain an appropriate pressure in the analysis compartment, all ABS samples (including fibers and 3D-printed samples) were outgassed overnight under ultra-high vacuum conditions prior to XPS analysis. The current investigation utilized the Ulvac PHI5000 Versa Probe high-resolution XPS spectrometer. The apparatus utilizes monochromatic Al Kα radiation (1486.7 eV) to stimulate the surface. The high-resolution spectra of C1s were recorded with a data pass energy of 23.5 eV and a 0.1 eV energy step. The main C1s peak was set to 285 eV.

#### 2.2.4. Fourier Transform Infrared Spectroscopy

ATR-FTIR is an effective technique used to analyze polymers [56,57,58,59,60]. The FTIR data were acquired using a Jasco FT/IR-4000 spectrometer from Tokyo, Japan. The examinations were conducted at ambient temperature with a resolution of 2 cm${\;\;}^{-1}$ across an entire range of 500–4000 cm${\;\;}^{-1}$. Prior to and following the 3D printing process, the polymer samples were assessed using ATR-FTIR spectroscopy. Prior to printing, the samples were in the form of filaments. However, after printing, the samples took the shape of cuboid objects.

#### 2.2.5. Differential Scanning Calorimetry (DSC)

The DSC curves were recorded on a Netzsch 200 F3 Maia device (Netzsch-Gerätebau GmbH, Selb, Germany) calibrated with indium. Around 11 mg of sample underwent a heating process in crucibles of aluminum with sealed shut and pierced lids. The curves were recorded at 10 °C min${\;\;}^{-1}$ and in nitrogen at a 50 mL min${\;\;}^{-1}$ flow rate.

#### 2.2.6. Broadband Dielectric Spectroscopy (BDS)

The dielectric assessments were performed using a broadband dielectric spectrometer (Novocontrol Technologies from Montabaur, Germany). Cylindrical round-shaped samples with diameters of 10 mm and thicknesses of 0.1 mm were prepared for BDS analyses. To ensure reliable ohmic contact, silver-conducting paint supplied by Novocontrol was applied to both the upper and lower surfaces of the samples. The samples were positioned between two plated flat electrodes (furnished by Novocontrol) and then introduced into the ZGS extension of the BDS instrument. Temperature control was maintained using a Quatro Cryosystem device. Dielectric data, encompassing dielectric permittivity and dielectric loss parameters, were acquired at a constant temperature, with frequency scans (ranging from 1 Hz to 1 MHz) taken every 5 °C within the temperature range of −150 °C to +150 °C. To avoid interference from moisture, the recordings were made in a dry nitrogen atmosphere.

#### 2.2.7. X-ray Diffraction

X-ray diffraction (XRD) is a good method utilized to discover and evaluate the crystalline structure of polymers. Its capacity to offer intricate details regarding the structure of a polymer makes it a compelling option for polymer characterization [61,62].

ABS specimens were analyzed for their nanoscale structure using a Rigaku Miniflex 600 diffractometer, which utilized the 1.54 Å of Cu Kα. The analysis was conducted in the 2θ range from 5.0 to 50.0°, with a 0.01° step and a recording rate of 1°/min.

#### 2.2.8. Mechanical Properties: Impact and Tensile Testing

A CEAST testing machine from CEAST, Italy, equipped with a 50 J energy pendulum, was used for Charpy impact properties of notched specimens (based on EN ISO 179-1:2023). A total of five specimens of every set of samples was tested (1 set for control, 1 set for the He–plasma-treated samples, 1 set for the Ar–plasma-exposed samples), each with a length of 80 mm, a width of 10 mm, and a thickness of 4 mm. The resulting average value was then reported.

Tensile studies were conducted using a tensile testing machine (Instron 3345 Single Column Systems, Norwood, MA, USA) with a 2.5 kN load cell for ABS specimens (based on ISO 527 1BA) [63]. For Young’s modulus estimation, the strain within the linear range was measured by a clip-on extensometer at a cross-head speed of 1 mm/min${\;\;}^{-1}$. Using a cross-head speed of 10 mm/min${\;\;}^{-1}$, both yield stress and elongation at break were recorded. A total of five tensile specimens, produced by additive manufacturing, were tested for each sample type: pristine ABS, helium–plasma-treated ABS, and argon–plasma-treated ABS.

All the samples were conditioned prior to mechanical testing for 24 h, at 50% relative humidity and 23 °C.

## 3. Results and Discussion

### 3.1. Electro-Optical Characterization of Plasma Source

The process of diagnosing plasmas at atmospheric pressure usually involves the observation and measurement of electro-optical parameters, which are then recorded and analyzed statistically [19,38,39,40,41,42,43,64].

**Figure 2:**
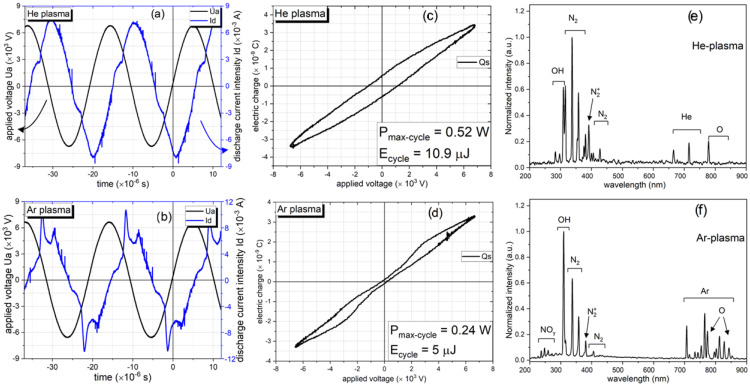
Typical voltage–current waveform of plasma in (**a**) He or (**b**) Ar. The Lissajous figure for (**c**) He–plasma and (**d**) Ar–plasma. The emitted spectra of the discharge in (**e**) He and (**f**) Ar.

The electrical diagnostic of the discharge was correlated with the measurement of applied voltage, discharge current intensity, charge monitoring, and the computation of power and energy. Figure 2a,b displays the typical voltage and current waveforms observed in plasma operating in helium and argon, respectively. The voltage–current representations achieved in these experiments are comparable to those obtained in prior tests performed under comparable settings [19,41,43].

The electrical power of He–plasma, calculated using Lissajous curves, varied between 0.1 and 0.52 W, with a equivalent energy range of 2 to 10.9 μJ. In the case of Ar–plasma, the power ranged from 0.05 to 0.24 W, with a corresponding energy range of 1 to 5 μJ. These values are shown in Figure 2c,d.

The plasma source used in this study has sufficient energy, as shown in Figure 2, to excite not only the spectral lines of the gas under study but also other atmospheric compounds, such as O, N${\;\;}_{2}^{+}$, OH, NO${\;\;}_{\gamma }$, N${\;\;}_{2}$, and N${\;\;}_{2}$, in addition to helium or argon lines. The plasma–surface interaction is significantly influenced by the so-called reactive species of nitrogen and oxygen (RNS and ROS).

The emitted spectra recorded at the Ar discharge–ABS interface mostly consist of the NO${\;\;}_{\gamma }$ lines, particularly at 237, 247, 259, and 271 nm, within the 200 to 300 nm wavelength range. The OH band was identified at 309 nm under both working gas conditions. Within the range of 315 to 390 nm, N${\;\;}_{2}$ was identified. The N${\;\;}_{2}^{+}$ seen at 391 nm is useful for determining the gas temperature via a Boltzmann plot. N${\;\;}_{2}$ reappears from 400 to 470 nm. The gas lines are seen between 580 and 740 nm for He–plasma and between 680 and 850 nm for Ar–plasma. Huzum et al. [41] and Nastuta et al. [19,42] assumed that the development and enhancement of N${\;\;}_{2}$ and N${\;\;}_{2}^{+}$ are dependent on the induced Penning effect by He metastables. Furthermore, the wavelengths of 777 and 845 nm were seen to exhibit spectral lines of atomic oxygen. Table 1 has a record of the spectral lines and bands seen under similar conditions, together with the ascribed transitions and the corresponding bonding energies E${\;\;}_{k}$ [65,66].

The dissociation of surrounding oxygen and water molecules is responsible for the existence of OH and O bands and lines in the emission spectra. This correlation has been established in previous experiments performed under similar plasma conditions [19,38,39,40,41,42,64].

The presence of reactive oxygen and nitrogen species with helium or argon lines, the working gases, suggests that they are involved in polymer treatment. Since they can be a measure of the gas vibrational and rotational temperatures, these highly excited species are valuable. Energy species employed in sample treatment can be determined from spectroscopic temperatures.

In addition, one can utilize simulation software such as Lifbase [67] and Spectrum Analyzer [68] to analyze the plasma-emitted spectra and determine some specific plasma temperatures. These temperatures include hydroxyl radicals’ rotational temperatures in addition to nitrogen molecular ions’ rotational temperatures. The rotational temperatures of OH and N${\;\;}_{2}^{+}$, frequently connected with the gas temperature (especially DBD-based sources), were observed to fluctuate from 338 to 357 K (65 to 83 °C), as previously reported by [40,41,42]. These values represented the ‘spectroscopic temperature’, which refers to the energies attainable by the OH and N${\;\;}_{2}^{+}$. These energies can be harnessed in the plasma environment and used to promote various reactions that occur either on the surface or within the volume of the plasma.

### 3.2. Surface and Volume Characterization Methods of Polymeric Sample

#### 3.2.1. Surface Morphology: Atomic Force Microscopy

**Figure 3:**
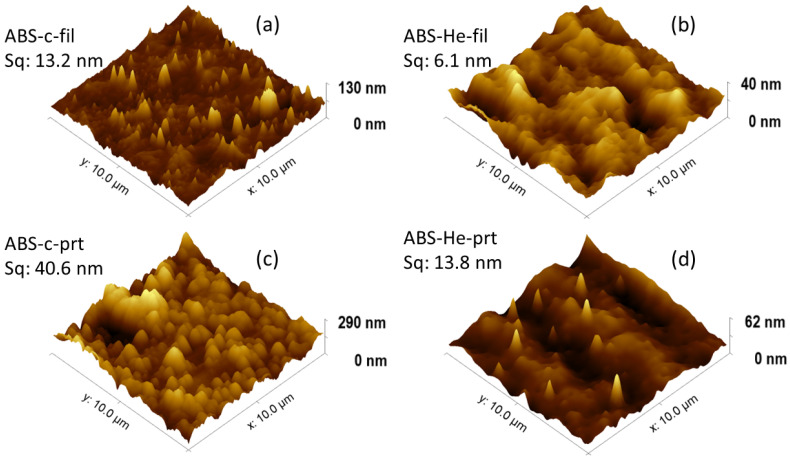
Topography of control (**a**) and He–plasma-treated filaments (**b**), as well as 3D-printed control (**c**) and He–plasma-treated (prt) samples (**d**).

AFM micrographs were acquired for every sample, with a 10 × 10 µm${\;\;}^{2}$ scanning area each. Imaging was carried out at multiple locations on the surface to certify the replicability of the findings. The results, related to three-dimensional images of the control and modified surfaces, are shown in Figure 3. AFM measurements were used to analyze and to compare surface texture by evaluating the root mean square roughness (S${\;\;}_{q}$) prior to and after the exposure. The value of the S${\;\;}_{q}$ of control ABS filaments is 13.2 nm, but for the control 3D-printed specimen, the value is 40.6 nm. The value of S${\;\;}_{q}$ is 6.1 nm for the plasma-modified filament and 13.8 nm for the plasma-exposed and 3D-printed sample. The 3D photos in Figure 3 clearly show notable changes in both the morphology and roughness of the ABS samples following plasma treatment, additive manufacturing, and the combined effect of both methods. This behavior may result from the rearrangement processes of ABS chains occurring on the polymer’s surface, which could involve crosslinking or the breaking of chemical bonds. The plasma treatment of the surface causes a smoothing process and the consistent restructuring of elevated points on the surface, enhancing the contact angle between ABS and whatever substance it encounters. This might enhance adherence on surfaces that were exposed to plasma. In order to validate such a supposition, additional characterization techniques such as CA, XPS, or FTIR are employed, as described in the subsequent sections of the text.

Subsequently, there is a notable proliferation of features apparent at the surface. Obviously, the increase in the S${\;\;}_{q}$ value, which is also contingent upon the duration of plasma exposure, is another manifestation of these effects. These results are summarized in Table 2, along other statistically meaningful surface quantities: S${\;\;}_{a}$, S${\;\;}_{sk}$, ek, S${\;\;}_{p}$, S${\;\;}_{v}$, and S${\;\;}_{z}$. By analyzing the morphological results shown in Figure 3 and considering the statistical values presented in Table 2, it may be stated that the plasma treatment had a smoothing effect on the nanoscale structures of the ABS polymer samples, specifically on the plasma-treated surfaces. By ‘smoothing’ the material, the interface between the substance and any solid, liquid, or vapor with which it may come into contact is increased.

Based on the statistical data calculated (Table 2) regarding the morphology of the studied ABS surfaces, we can conclude that plasma treatments result in a consistent decrease in value of the S${\;\;}_{q}$, as well as in S${\;\;}_{p}$ and S${\;\;}_{v}$, for both filaments and 3D-printed samples. For S${\;\;}_{q}$, a reduction of 55% for filaments and 66% for printed samples is observed following exposure to plasma; for S${\;\;}_{p}$, a decrease by 33% for filaments and 67% for printed samples is observed; and for S${\;\;}_{v}$, a drop in the value by 47% for filaments and 68% for printed samples is observed. These values suggest a significant reorganization of the studied surfaces, with quite a leveling effect, as shown in the topography images in Figure 3.

#### 3.2.2. Static CA and Surface Energy

**Figure 4:**
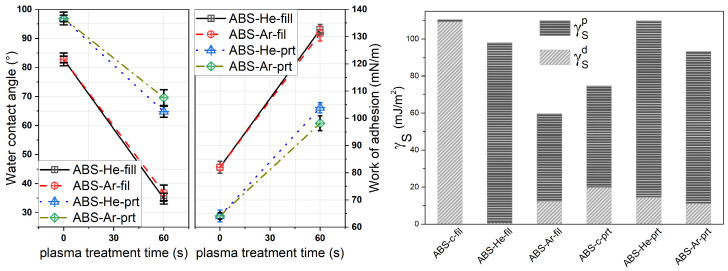
Water CA and Wa values obtained for the ABS (**left graph**) and the surface energy (**right graph**) measurements of control and He/Ar–plasma-treated filament (fill) and printed (prt) samples.

The quantitative data of CA and work of adhesion (Wa) for the ABS specimens are shown in the left panel of Figure 4, and the surface energy is displayed in the right panel. The data points are the averages determined from at least three different measurements, with a standard deviation of ±2 degrees. The polar and dispersive components of distilled water and glycerol were used to determine the value of surface free energy ($\gamma_{S}$). Additionally, the values for the surface tensions ($\gamma_{L}$) of test liquids were used to evaluate the $\gamma_{S}$ values of ABS specimens and its components, namely polar ($\gamma_{S}^{p}$) and dispersive ($\gamma_{S}^{d}$), through the Owens, Wendt, Rabel, and Kaelble (OWRK) model, as described by Good [69,70,71]. At 23 °C, $\gamma_{Lwater}$ = 72.80 mN/m and $\gamma_{Lglycerol}$ = 64 mN/m, as reported also in previous work [5,19]. The CA values, Wa, relative increase in adhesion work (as described by Birleanu et al. [5]), γ, and polar index are given in Table 3.

The CA value of the control ABS filament is 82° for distilled water and 69° for glycerol. Post-He-discharge exposure, the CA values decrease to 34° for water and 47° for glycerol. In the case of Ar-discharge treatment, the CA drops to 36° for water and 38° for glycerol (as shown in Figure 4). This is a clear illustration of surface functionalization. As shown in Table 3, the 3D-printed polymer samples exhibited a comparable pattern, exhibiting a small reduction in CA value for both liquids.

Over the course of the experiments, it was discovered that the tendency to reduce the contact angle value is maintained not only for filaments but also for the 3D-printed samples; however, there was a lower influence on the latter. Considering that the process of 3D printing includes the polymer being heated and cooled in a series of steps, it is logical to suppose that reorganizations of the polymer chains occur throughout the process. Moreover, following the He–plasma and Ar–plasma treatments, a clear improvement in the surface energy is observed through the work of adhesion size. In addition, the dispersed and polar indices display an evolution in the sense of the plasma exposure of the surfaces. More precisely, an increase in the polar component is experimentally found; following the exposure of the polymer material in plasma, the effect is enhanced in the case of He–plasma treatment for ABS filaments. There is a slight shift in favor of treatments conducted in argon plasma for 3D-printed materials. Table 3 presents the trends in the values of the parameters related to adhesion, surface free energy, and dispersion and polar indices. Based on the determined values, a reduction in the CA value of 55% was seen for the filament samples and 30% was seen for printed samples after plasma treatment. The work of adhesion reveals a relative increase by 60%.

#### 3.2.3. XPS Spectroscopy

**Figure 5:**
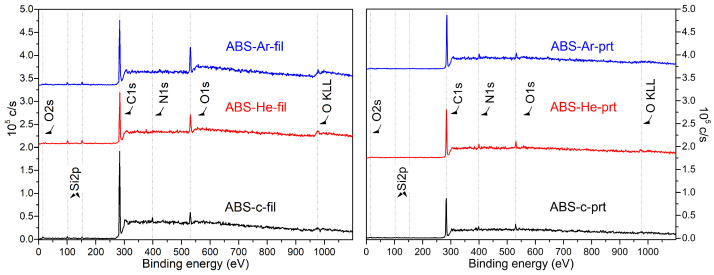
XPS wide spectrum of the ABS samples in filament sate (‘fil’, **left panel**) and in printed state (‘prt’, **right panel**) for untreated (control ‘c’) and He–plasma- and Ar–plasma-treated specimens.

The main peaks for ABS are for C1s (285 eV), N1s (399 eV), and O1s (531 eV), as depicted in Figure 5.

ABS is a polymer that lacks oxygen in its chemical structure. However, there have been frequent reports of the presence of oxygen near the surface, which may be accredited to additives utilized in the copolymer’s manufacturing process as well as airborne contaminants, including adsorbed water or CO${\;\;}_{2}$ [72].

The quantitative study of the untreated surface, using peak areas, provides the following values for the ABS filament samples: 91.86% for C1s, 5.78% for O1s, and 2.36% for N1s. These values are similar to those reported by Wang et al. [73] but slightly different from the percentages reported in the literature for virgin polymers (C 86%, O 12%, and N 2%) by [72,74]. So, after treating the ABS samples, the strength of the peak associated with C–H and C–C reduced, whereas the intensity of the signal related to N and O increased. Similar data reported by Ohkubo et al. [74] suggest that the plasma treatment may dissolve butadiene rubber and could add oxygen-containing functional groups.

Table 4 displays the elemental composition of ABS specimens as determined by wide-scan spectra. It can be observed that the atomic ratio, calculated from the wide spectra, changes upon discharge exposure. The increase in the O and N content support the idea of increased surface adhesion.

Based on the acquired data, it was concluded that the plasma exposure of ABS surfaces led to an enhanced atomic fraction of O and N content. Conversely, a decrease in the relative atomic fraction of C (Table 4) was observed. At the same time, the surface O/C ratios escalate with plasma exposure for the filament samples, while for the printed samples, only for the He–plasma-exposed samples was the increase was observed, and for the Ar–plasma-exposed samples, a decrease was seen. The N content showed a slight increase after the He–plasma treatment of the filament sample, while for the rest of the conditions, the N content decreased.

It is important to emphasize that the carbon content on the polymer surface does not decrease after plasma treatment. Instead, the oxygen and nitrogen contents increase near the plasma–polymer interface. This is a result of the adsorption of water and nitrogen onto the surface of the polymer, as well as their existence between the electrodes at ambient pressure. This has been confirmed through optical emission spectroscopy measurements (see Figure 2e,f). ROS and RNS interact with the surface, increasing the amount of oxygen and nitrogen while reducing the carbon bonds, as shown in the XPS measurements. The XPS results indicate that after plasma exposure, there is an enhancement in the presence of oxygen-containing groups, accompanied by a decrease in the main C–C bonds in all samples. This occurs as a result of the plasma causing the main carbon-containing bonds to break, resulting in the creation of hydroxyl, carboxyl, and carbon-to-oxygen double bonds at normal atmospheric pressure. This also serves as evidence that the surface becomes more capable of being wetted after undergoing plasma therapy [40]. Higher oxygen content seen in the case of Ar–plasma treatment, as presented in Table 4, is due to the surface oxidation of ABS fibers. Specifically, there is up to a 15.85% increase in O1s content and up to a tripling of O/C content. Plasma treatment involving atmospheric pressure plasmas leads to the occurrence of chain-propagating radical reactions, which are caused by ROS and RNS. These reactions can result in the creation of many oxidation products, such as alcohols and carbonyl compounds like carbonyls, ketones, aldehydes, carboxylic acids, ethers, and even esters. These oxidation products are likely to interact with the polymer surface, resulting in its modification, usually related to enhanced surface adhesion.

#### 3.2.4. ATR-FTIR Spectroscopy

**Figure 6:**
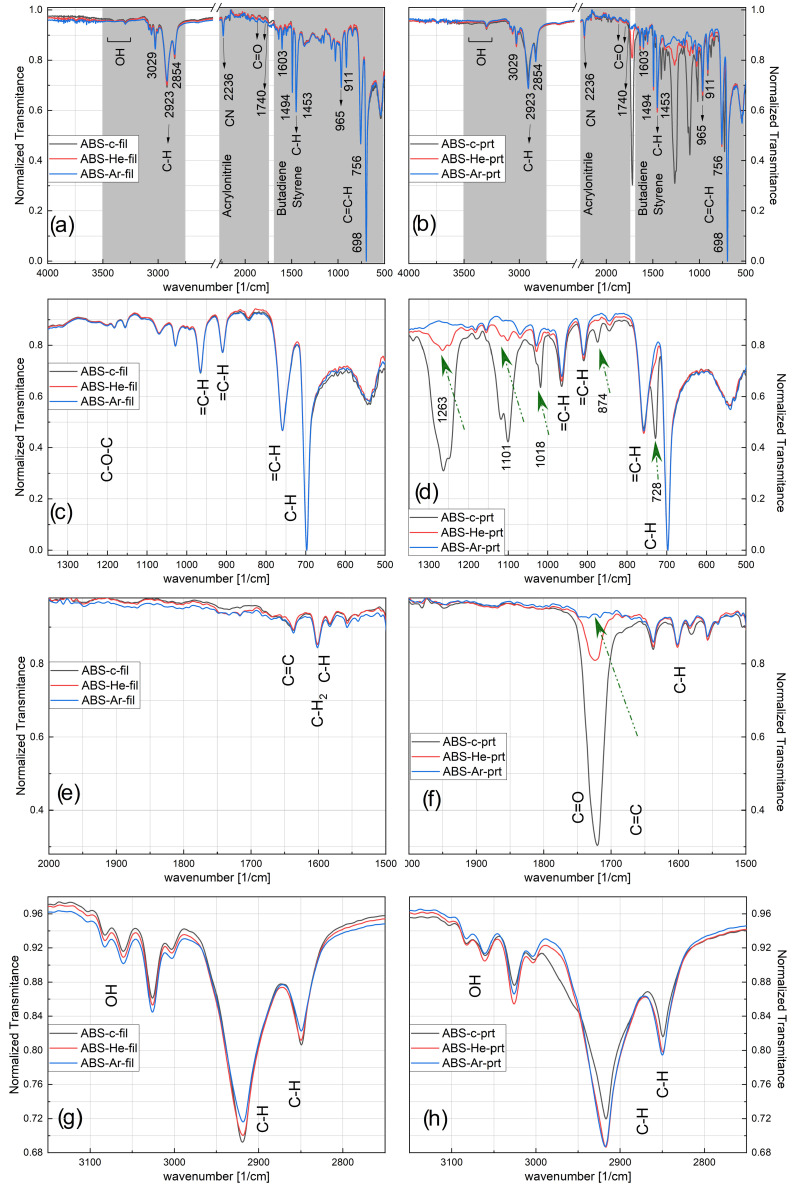
The ATR-FTIR spectra of the ABS specimens, with the filament samples (fil: **a**,**c**,**e**,**g**) on the left side and the printed samples (prt: **b**,**d**,**f**,**h**) on the right side.

The higher wettability of the filament exposed to plasma and the 3D-printed specimen may also be detected by ATR-FTIR. Figure 6 displays the ATR-FTIR spectrum for untreated ABS and ABS treated with He–plasma and Ar–plasma. The spectra are displayed for both the filament (‘fil’ left column of Figure 6) and after 3D printing (‘prt’ right column of Figure 6). Most of the bands found in the ABS spectra are presented in Table 5. Besides the bands assigned to the ABS copolymer, we also find bands assigned to the aromatic bend of C–H and C=H, the vibration of C–O–C, CH${\;\;}_{2}$ bending, stretching overtone, and symmetric and asymmetric stretch.

However, even if the studied polymer does not contain native O, such bands containing oxygen are found and identified due to the plasma treatment and exposure to the environment. As in graphs from Figure 6a,b, the material identification elements are present, confirming the authenticity of the material used as being ABS through its constituent components: the band at wave number 1494 cm${\;\;}^{-1}$ attributed to styrene, the one at 1637 cm${\;\;}^{-1}$ attributed to butadiene, and the band at wave number 2238 cm${\;\;}^{-1}$ assigned to acrylonitrile [1,35,75,76,77].

It should be noted that there is a general tendency to increase the intensity of the majority of the peaks after plasma treatments, both for the filament-type samples (Figure 6a,c,e,g) and for the 3D-printed ones (Figure 6b,d,f,h). Moreover, for ABS filaments, a rise in the assigned OH band is observed after plasma treatments, and a more pronounced effect is observed for the Ar–plasma exposure (Figure 6g).

In the case of the printed samples, one can also observe the disappearance of some bands after exposure to the plasma, such as the band at wave number 728 cm${\;\;}^{-1}$, as well as those at 874, 1018, 1101, and 1263 cm ${\;\;}^{-1}$ (Figure 6d) and 1721 cm${\;\;}^{-1}$ (Figure 6f).

The results suggests that upon He–plasma exposure, the ABS surface interacts with an enhanced number of ROS, resulting in subtle alterations in the C–O and COOH regions. There are also some changes detected in the ABS when exposed to argon plasma, yet they are less evident. However, the findings indicate that, in the specific circumstances outlined here, plasma exposure mainly impacts the surface of the material rather than its inner structure. In addition, the process of additive manufacturing can be considered a form of annealing, which results in an increase in the level of crystallinity of the polymers.

Nevertheless, the enduring impact of the plasma treatments on the printed polymer suggests that their influence remains significant throughout the additive manufacturing procedure.

#### 3.2.5. Differential Scanning Calorimetry (DSC)

**Figure 7:**
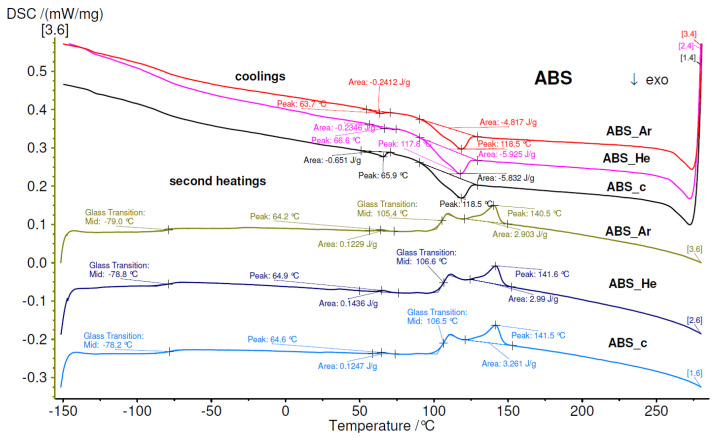
DSC thermograms of the ABS samples.

The DSC thermograms of the ABS samples are shown in Figure 7. Typically, the DSC data were recorded over two heating cycles and one intermediate cooling phase within temperatures from −150 °C to 280 °C. The first heating was used to wipe out the thermal history of the samples. Subsequent cooling and second heating cycles (depicted in Figure 7) were further collected to assess the calorimetric events of the ABS samples.

In the second heating thermogram of the pristine ABS, the glass transition temperature (T${\;\;}_{g}$) as well as the melting temperature (T${\;\;}_{m}$) are observed to be 107 °C and 142 °C, respectively. The T${\;\;}_{g}$ at 107 °C can be associated with the polystyrene component, while the T${\;\;}_{m}$ at 142 °C is dependent on the acrylonitrile component [78]. Notably, for plasma–treated samples, these two thermal events occur at lower temperatures (specific values are indicated in Table 6). On the cooling curve, the crystallization of ABS samples is indicated by an exothermal peak (T${\;\;}_{c}$) at 119 °C, with no significant variations between pristine and plasma–treated samples.

From Figure 7, it can also be observed that the DSC thermograms contain additional signals. For instance, the thermograms of pristine ABS enclose a step increase at −78 °C and an endothermal peak at 65 °C during the second heating curve, along with an exothermal peak at 66 °C in the cooling curve. These signals can be associated with the T${\;\;}_{g}$, T${\;\;}_{m}$, and T${\;\;}_{c}$, which can be attributed to the polybutadiene component [78]. The thermograms of plasma–treated samples exhibit similar thermal characteristics.

#### 3.2.6. Dielectric Properties

**Figure 8:**
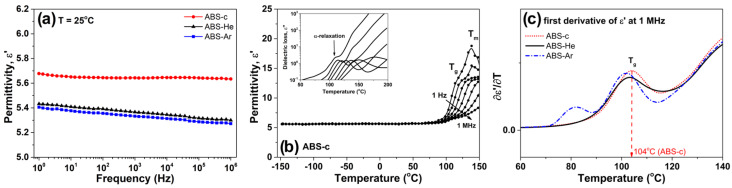
(**a**) The evolution of $\epsilon^{\prime }$ with frequency for ABS samples at 25 °C. (**b**) The evolution of $\epsilon^{\prime }$ with temperature at various frequencies for ABS-c sample. The inset shows the evolution of $\epsilon^{\prime \prime }$ with temperature at various frequencies for ABS-c sample. (**c**) Comparative evolution of the first derivative of $\epsilon^{\prime }$ with temperature at a frequency of 1 MHz for ABS samples. In panels (**b**,**c**), the signals corresponding to the glass transition temperature (T${\;\;}_{g}$) and melting temperature (T${\;\;}_{m}$) highlight the dielectric characteristics.

Figure 8 illustrates the frequency dependencies of the dielectric permittivity ($\epsilon^{\prime }$) for ABS samples. The broadband dielectric spectroscopy (BDS) spectra are collected at T = 25 °C. $\epsilon^{\prime }$ exhibits relatively high values (e.g., for untreated ABS at f = 1 kHz, $\epsilon^{\prime }$ = 5.6) and experiences a slight decrease with increasing frequency. For the ABS samples exposed to plasma, the magnitude of $\epsilon^{\prime }$ is noticeably diminished across the entire frequency range, indicating a restrained activity of molecular dipole moments.

The temperature dependencies of $\epsilon^{\prime }$, selected at different frequencies, are given in panel b of Figure 8, with the ABS-c sample as the exemplar. The other investigated samples show similar tendencies. At temperatures of ABS below T${\;\;}_{g}$, $\epsilon^{\prime }$ increases slowly with temperature (e.g., for ABS–He, $\epsilon^{\prime }$ = 5.3 at −150 °C and 5.8 at 90 °C at a frequency of 1 Hz). In this region, limited fluctuations in the permanent dipoles from ABS are sensitive to the temperature changes. On the other hand, at temperatures higher than 100 °C, a noticeable stepwise increase in $\epsilon^{\prime }$ is detected. The inset in Figure 8b presents the evolution of $\epsilon^{\prime \prime }$ with temperature at various frequencies for the ABS-c sample. The $\epsilon^{\prime \prime }$(T) dependencies reveal the presence of the dipolar α-relaxation process, which is closely connected with the glass transition temperature of the material. Accordingly, with DSC findings, the increased magnitudes of $\epsilon^{\prime }$ and $\epsilon^{\prime \prime }$ parameters originate from the segmental motions of the polystyrene component of ABS. The dielectric dispersion of the permittivity function is also affected by the melting point of the acrylonitrile component of ABS. We should note that the glass transition temperature determined through dielectric spectroscopy measurements appears slightly lower compared to that obtained through DSC. This variance can be explained by the distinct heating rates employed in BDS and DSC techniques. DSC experiments utilized a heating rate of 10 °C min${\;\;}^{-1}$, whereas BDS measurements operated at a considerably slower heating rate of around 1 °C min${\;\;}^{-1}$. The latter scenario results in T${\;\;}_{g}$ values being assessed at lower temperatures. The glass transition point of ABS samples may be assessed using dielectric spectroscopy by evaluating the dielectric permittivity function’s first derivative [79]. The isochronal plots of the first derivative function versus temperature are given in Figure 8c for all considered samples. The peak maxima were associated with the dielectric glass transition and presented in Table 7. It is worth noticing that the dielectric T${\;\;}_{g}$ of He–plasma- and Ar–plasma-treated samples are slightly diminished, as compared with that of untreated plasma ABS material. Similar results were found for calorimetric measurements. The decrease in T${\;\;}_{g}$ for plasma-treated samples may suggest that the segmental motions of the ABS backbone are slightly promoted.

The dielectric constant of a substance is inversely related to the material’s density. A potential reduction in the $\epsilon^{\prime }$ value may result from the heightened polymer density during plasma exposure and subsequent 3D printing, leading to a more condensed printed object with a lower dielectric constant. Simply said, when the density rises, the dielectric constant decreases.

#### 3.2.7. X-ray Diffraction

X-ray diffraction studies were performed to assess the phase crystallization of ABS objects created using 3D-printing technology. The formation of the amorphous phase of the copolymer is evidenced by a prominent peak in pure ABS, located at a diffraction angle of around 20°, as well as from the presence of a smaller peak at around 13°, as reported by [76,77,80,81,82]. The crystallinity index of pure ABS, which is primarily amorphous, is influenced by the small peaks that emerge during plasma treatment and the 3D-printing process.

The degree of crystallinity (or crystallization capacity) X${\;\;}_{c}$ is an important indicator for polymers undergoing processing. It is determined by analyzing the diffractograms of the polymeric samples, based on the ratio between the area of crystalline peaks and the sum of all crystalline and amorphous peaks. This is achieved by analyzing the amorphous and crystalline components of the diffraction intensity.

**Figure 9:**
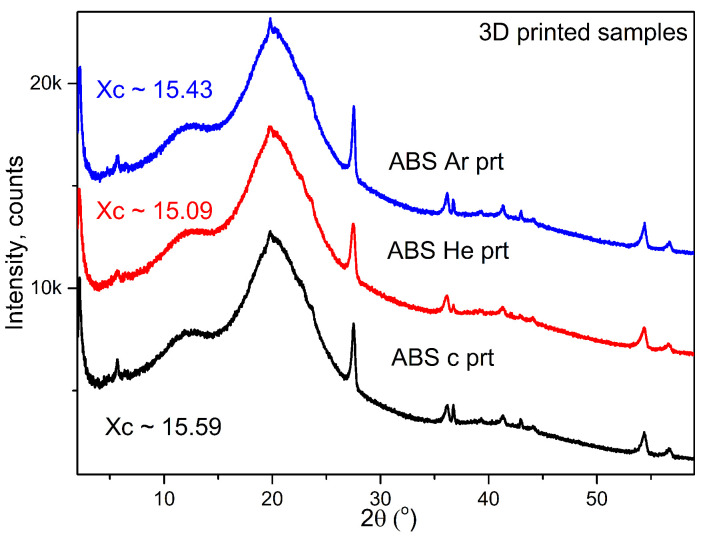
Diffractograms of studied 3D-printed polymers: control (c), He–plasma-, and Ar–plasma-treated ABS.

XRD studies have shown that ABS filaments usually have a granular crystalline structure. 3D printing results in a more compact structure with polymerized domains that have a reduced degree of crystallinity. It should be noted that 3D-printing materials can create air gaps between layers of filaments. Improved filament layer connectivity and reduced material shrinkage result in narrower gaps. The XRD spectra of the ABS 3D-printed products may be found in Figure 9 (according to [83]).

#### 3.2.8. Mechanical Properties: Impact and Tensile Testing

Mechanical properties are important to the design materials for different applications. The impact test quantifies the energy absorbed by a sample during fracture, while impact energy measures the work required to fracture a test sample. Usually, in practice, the breaking strength is used. Just as tensile strength measures the maximum force a material can endure before fracturing, break strength indicates the threshold at which a material may fracture when subjected to rapid force or stress. It is generally recognized that 80% of the tensile strength represents the breaking strength. Young’s modulus is a constant value that characterizes the elastic behavior of a solid material under tension. The property being described is the sample’s ability to resist variations in length, sometimes referred to as the modulus of elasticity. Elongation at break, or fracture strain, is the ratio of the modified length to the initial length after the test specimen breaks. It demonstrates a material’s capacity to withstand deformation without developing cracks.

As reference values, the manufacturer of the ABS filament (Devil Design Sp. J., Poland) states that the tensile strength is 42 MPa, while the value of the tension elongation at break is around 30%. The mechanical properties of the studied ABS printed samples are displayed in Table 8. It includes information regarding impact strength, impact energy, Young’s modulus, breaking strength, and elongation at break.

The results obtained highlight a different mechanical behavior of the ABS samples subjected to the plasma treatment. Thus, after exposure to He–plasma, we observe a decrease in impact strength by 7.8%, while the samples exposed to Ar–plasma recorded an increase of 6% in impact strength. A similar behavior was observed for impact energy: a decrease of 4.2% was observed for the samples exposed to He–plasma, and an increase of 6.2% was observed for those exposed to Ar–plasma. Both He–plasma and Ar–plasma exposure resulted in a decrease in tensile properties. Thus, Young’s modulus decreased by 0.45% for samples exposed to He–plasma and by 1.22% for those exposed to Ar–plasma. Based on the results obtained, we can state that, as compared to the values specified by the ABS filament manufacturer, the values of the tensile strength parameter were 83.8% for the untreated 3D printed ABS sample, 80% for the ABS He–plasma-treated samples, and 78.1% for the ABS Ar–plasma-treated samples. A decrease in tensile strength of 3.8% and 5.7% was revealed for the treated samples, both in He–plasma and Ar–plasma respectively. These results suggest a decrease in the tensile strength after plasma treatment of the samples. The elongation at break was slightly improved after plasma treatments. Thus, after exposure to He–plasma, an increase of 13.5% was observed, while the exposure to Ar–plasma resulted in an increase of 21.6% compared to the untreated samples. Similar trends were obtained in the literature under similar conditions for both impact testing [77,81,84,85,86] and tensile tests [77,81,84,87,88,89,90,91,92,93,94].

**Figure 10:**
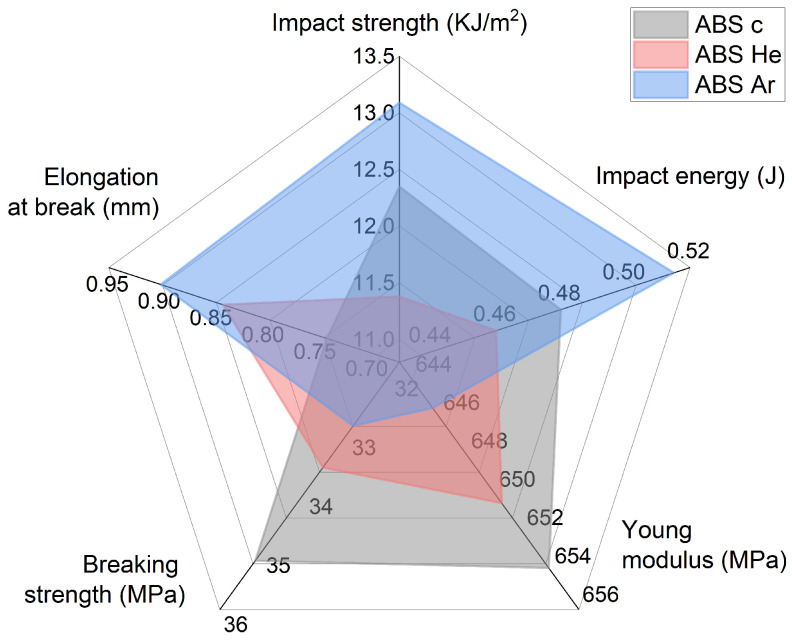
The spider diagram illustrates the mechanical characteristics of the 3D-printed polymer samples being tested. The samples include untreated ABS (depicted by grey region), ABS treated with helium plasma (expressed by the red area), and ABS treated with argon plasma (represented by the blue area).

Figure 10 displays a spider diagram illustrating the mechanical characteristics of the studied 3D-printed polymer sepcimens: untreated (represented by the grey area), He–plasma-treated (represented by the red area), and Ar–plasma-treated (represented by the blue area) ABS. The mechanical features of the samples can be observed to clump together based on the plasma treatment they received. Furthermore, the results obtained for each sample indicate that the Ar–plasma-treated sample exhibits greater stability during impact tests and elongation at break, as shown in Figure 10. On the other hand, the He–plasma-treated samples demonstrate superior performance in terms of breaking strength and Young’s modulus compared to the other samples.

## 4. Conclusions

Examining plasma sources uncovers operational properties for manipulating surfaces through the use of electrical and optical diagnostic techniques. He–plasma has an electrical power ranging from 0.1 to 0.52 W, with a 2 to 10.9 μJ energy range. Ar–plasma has a power range of 0.05 to 0.24 W, with 1 to 5 μJ energy range. Moreover, based on emission spectroscopy observations, there were identified ROS and RNS species that are beneficial to starting physico-chemical reactions at the interface with polymers.

Plasma treatment transforms ABS polymer samples into homogeneous nanoscale structures. Roughness decreases by 55% for filament samples and 65% for printed ones following plasma treatment. This morphology is affected by ABS chain restructuring on the polymer’s surface. Plasma treatment levels the nanoscale structures of ABS polymer samples.

Plasma exposure reduced contact angle by 55% (for both water and glycerol) and increased adhesion by up to 60% for ABS filaments. The printed specimens showed a lower but constant 30% reduction in CA value. Additionally, the polar index increased from 0.01 to 0.99 for He–plasma-treated filament and from 0.73 to 0.86 for printed samples. A similar tendency, but with slightly lower values, was found for the Ar–plasma-treated filament and 3D-printed samples.

XPS experiments show that plasma exposure increases oxygen-containing group concentration in ABS filament samples. In plasma-treated filament samples, O content increased by 10% after He–plasma exposure and by 13% after Ar–plasma exposure. In 3D-printed samples, alterations were lower, but in He-treated samples, they increased from 4.7 to 5.8%. The combined WCA and XPS findings suggest that DBD plasma treatments modify ABS sample surface energy.

The DSC thermograms of the plasma-treated materials exhibited comparable thermal properties, with a slight decrease. The samples treated with plasma exhibited slightly reduced T${\;\;}_{g}$ and T${\;\;}_{m}$ occurrences at around 107°.

Based on the BDS measurements, He- and Ar-treated samples had lower dielectric T${\;\;}_{g}$ than untreated plasma ABS material at around 104°. The dielectric constant falls with material density, possibly due to plasma exposure and 3D-printing increasing polymer density. From the DSC and BDS data, it can be concluded that after plasma treatment, for ABS, the glass temperature can decrease. This means that, as future application, the manipulation of the 3D processing of this material could be performed at a lower temperature, which could result in less expensive processing.

XRD results showed only small variations, except for a few extra peaks that may be connected with a modest glass temperature change recorded by DSC measurements. Pure ABS exhibits the amorphous phase with a large peak at 19° and a smaller peak at 13°. The crystallinity index was approximately 15% across all samples.

Plasma-treated ABS samples have different mechanical properties. He–plasma decreased impact strength by 7.8%, whereas Ar–plasma increased it by 6%. He–plasma and Ar–plasma exposures lowered Young’s modulus by 0.45% and 1.22%, respectively. Tensile strength values dropped by 3.8% and 5.7%, respectively. He–plasma exposure increased break elongation by 21.6%. Similar tendencies were found in the literature.

As a a general remark, for the conditions used in the present study, the He–plasma treatment seems more effective on ABS samples than the Ar–plasma treatment. Moreover, additional research is required to adequately regulate the surface alterations induced by plasma treatment prior to their successful management. Aside from scratch and pull-off tests, further examinations such as ink testing, three-point bending, and any other applicable techniques will be considered.

## Figures and Tables

**Table 1:** Spectral lines and bands, with energies E${\;\;}_{k}$ from [65,66], that were detected in the plasma.

λ [nm]	Transition	E${\;\;}_{k}$ [eV]
308 OH	$\left(A^{2}\Sigma^{+}\right)$←$\left(X^{2}\Pi_{i}\right)$	4
337 N${\;\;}_{2}$	${\left(C^{3}\Pi_{u}\right)}_{\nu^{\prime }=0}$←${\left(B^{3}\Pi_{g}\right)}_{\nu^{\prime \prime }=0}$	11.0
391 N${\;\;}_{2}^{+}$	${\left(B^{2}\Sigma_{u}^{+}\right)}_{\nu^{\prime }=0}$←${\left(X^{2}\Sigma_{g}^{+}\right)}_{\nu^{\prime \prime }=0}$	18.7
587 He	(3d)←(2p)	23.1
667 He	(3d)←(2p)	23.1
696 Ar	(4s)←(4p)	13.3
706 He	(3s)←(2p)	22.7
728 He	(3s)←(2p)	22.9
772 Ar	(4s)←(4p)	13.3
777 O	3sS205←3pPi5	10.7
811 Ar	(4s)←(4p)	13.0
826 Ar	(4s)←(4p)	13.3
844 O	3sS103←3pPi3	11.0

**Table 2:** AFM statistical quantities for polymer samples under investigation.

Sample	S${\;\;}_{q}$ (nm)	S${\;\;}_{a}$ (nm)	S${\;\;}_{\mathrm{sk}}$	ek	S${\;\;}_{p}$ (nm)	S${\;\;}_{v}$ (nm)	S${\;\;}_{z}$ (nm)
ABS${\;\;}_{c}$-fil	13.25	9.74	1.13	3.48	95.32	42.53	137.86
ABS${\;\;}_{He}$-fil	6.10	4.58	0.79	4.81	63.46	22.48	85.94
ABS${\;\;}_{c}$-prt	40.6	43.84	0.17	0.32	178.66	178.16	356.82
ABS${\;\;}_{He}$-prt	13.80	10.86	0.20	0.35	58.68	55.95	114.63

**Table 3:** ABS samples’ contact angles, relative increases in work of adhesion, and surface free energies.

	Contact Angle		Work of Adhesion		$\Delta W_{a}/W_{a}$ ^1^		Surface Free Energy			$x^{p}$ ^2^
	(°)		(mN/m)		(%)	(%)	(mN/m)			(%)
Sample	Distilled Water	Glycerol	W_12_-Water	W_12_-Glycerol	Water	Glycol	***γ_S_***	***γ_S_^d^***	***γ_S_^p^***	***γ^p^*/*γ***
ABS${\;\;}_{c}$-fil ^3^	82	69	81.96	86.06	–	–	110.61	109.40	1.21	0.010
ABS${\;\;}_{He}$-fil	34	47	132.56	107.61	61.74	25.03	98.04	0.78	97.25	0.991
ABS${\;\;}_{Ar}$-fil	36	38	131.10	114.17	59.96	32.65	59.76	12.13	47.63	0.797
ABS${\;\;}_{c}$-prt ^4^	96	107	64.10	44.90	–	–	74.65	19.86	54.79	0.733
ABS${\;\;}_{He}$-prt	64	76	103.88	78.80	62.04	75.52	109.86	14.52	95.34	0.867
ABS${\;\;}_{Ar}$-prt	69	80	98.17	74.93	53.14	66.89	93.31	11.2	82.11	0.879

^1^ $\Delta W_{a}/W_{a}$ = relative increase in the adhesion work. ^2^ x${\;\;}^{p}$(%) = polar index. ^3^ fil = ABS filament. ^4^ prt = printed ABS.

**Table 4:** Atomic ratio of control and plasma-treated ABS surfaces.

Sample	Measured Atomic Ratio (%)			O/C Ratio (%)	N/C Ratio (%)
	C1s	O1s	N1s		
ABS${\;\;}_{c}$-fill ^1^	91.86	5.78	2.36	6.29	2.56
ABS${\;\;}_{He}$-fill	83.54	13.64	2.82	16.32	3.37
ABS${\;\;}_{Ar}$-fill	82.7	15.85	1.45	19.16	1.75
ABS${\;\;}_{c}$-prt ^2^	89.81	4.29	5.9	4.77	6.56
ABS${\;\;}_{He}$-prt	89.79	5.28	4.93	5.88	5.49
ABS${\;\;}_{Ar}$-prt	90.95	3.87	5.18	4.25	5.69

^1^ fill = ABS filament. ^2^ prt = printed ABS.

**Table 5:** Characteristic wavenumbers (cm${\;\;}^{-1}$) for ABS samples, as reported by [1,35,75,76,77].

Wavenumber Regions (cm${\;\;}^{-1}$)	Groups Assignment
698–699	Aromatic C–H bend
759	Aromatic C–H bend and =C–H bend
911–967	=C–H bend in poly(butadiene)
1215	C–O–C vibrations
1452–1453	CH${\;\;}_{2}$ bend/scissoring mode
1494–1497	Aromatic ring in styrene
1583	Aromatic ring in styrene
1600–1900	O–H stretching; C–H${\;\;}_{3}$, C–H${\;\;}_{2}$, and C–H stretch first overtones
1602	Aromatic ring in styrene
1637	C=C stretch mode of poly(butadiene)
1718	C=O stretch
1781–1860	C=O asymmetric and symmetric stretch from anhidride groups
2237–2239	CN stretching from acrylonitrile
2800–3127	C–H stretching from aromatic and aliphatic
3300	ABS additives
3000–3500	–OH water

**Table 6:** Thermal data extracted from DSC measurements.

Sample	T${\;\;}_{g}$ (°C)	T${\;\;}_{m}$ (°C)	T${\;\;}_{c}$ (°C)
ABS${\;\;}_{c}$	107	142	119
ABS${\;\;}_{He}$	107	142	118
ABS${\;\;}_{Ar}$	105	141	119

**Table 7:** Values of the dielectric permittivity and the dielectric glass transition evaluated from BDS measurements. The $\epsilon^{\prime }$ values were selected at 1 Hz and 25 °C. The dielectric T${\;\;}_{g}$ was assessed using the isochronal plots.

Sample	$\epsilon^{\prime }$	T${\;\;}_{g}$ (°C)
ABS${\;\;}_{c}$	5.7	104
ABS${\;\;}_{He}$	5.4	103.2
ABS${\;\;}_{Ar}$	5.4	101.7

**Table 8:** Mechanical properties of the 3D-printed ABS samples for impact (Charpy) and tensile tests.

Sample	Impact Strength	Impact Energy	Young’s Modulus	Tensile Strength	Elongation at Break
	(kJ/m^2^)	(J)	(MPa)	(MPa)	(mm)
ABS${\;\;}_{c}$	12.35 ± 1.5	0.48 ± 0.06	653 ± 20	35.17 ± 0.8	0.74 ± 0.01
ABS${\;\;}_{He}$	11.38 ± 1.1	0.46 ± 0.04	650 ± 20	33.59 ± 1.6	0.84 ± 0.08
ABS${\;\;}_{Ar}$	13.09 ± 1.4	0.51 ± 0.05	645 ± 15	32.88 ± 0.6	0.90 ± 0.04

## Data Availability

Raw data may be available, on reasonable request, from the authors.
